# The multiple effects of *REG1* deletion and *SNF1* overexpression improved the production of *S*-adenosyl-l-methionine in *Saccharomyces cerevisiae*

**DOI:** 10.1186/s12934-022-01900-7

**Published:** 2022-08-27

**Authors:** Hailong Chen, Xiaoqin Chai, Yan Wang, Jing Liu, Guohai Zhou, Pinghe Wei, Yuhe Song, Lingman Ma

**Affiliations:** 1 Jiangsu Key Laboratory of Chiral Pharmaceuticals Biosynthesis, College of Pharmacy and Chemistry & Chemical Engineering, Taizhou University, 93 Ji Chuan Road, 225300 Taizhou, Jiangsu People’s Republic of China; 2 School of Life Science and Technology, China Pharmaceutical University, 211198 Nanjing, Jiangsu People’s Republic of China

**Keywords:** *Saccharomyces cerevisiae*, *S*-adenosyl-l-methionine, Glucose repression, Lifespan, *SNF1*, *REG1*

## Abstract

**Background:**

*Saccharomyces cerevisiae* is often used as a cell factory for the production of S-adenosyl-l-methionine (SAM) for diverse pharmaceutical applications. However, SAM production by *S. cerevisiae* is negatively influenced by glucose repression, which is regulated by a serine/threonine kinase SNF1 complex. Here, a strategy of alleviating glucose repression by deleting *REG1* (encodes the regulatory subunit of protein phosphatase 1) and overexpressing *SNF1* (encodes the catalytic subunit of the SNF1 complex) was applied to improve SAM production in *S. cerevisiae*. SAM production, growth conditions, glucose consumption, ethanol accumulation, lifespan, glycolysis and amino acid metabolism were analyzed in the mutant strains.

**Results:**

The results showed that the multiple effects of *REG1* deletion and/or *SNF1* overexpression exhibited a great potential for improving the SAM production in yeast. Enhanced the expression levels of genes involved in glucose transport and glycolysis, which improved the glucose utilization and then elevated the levels of glycolytic intermediates. The expression levels of *ACS1* (encoding acetyl-CoA synthase I) and *ALD6* (encoding aldehyde dehydrogenase), and the activity of alcohol dehydrogenase II (ADH2) were enhanced especially in the presence of excessive glucose levels, which probably promoted the conversion of ethanol in fermentation broth into acetyl-CoA. The gene expressions involved in sulfur-containing amino acids were also enhanced for the precursor amino acid biosynthesis. In addition, the lifespan of yeast was extended by *REG1* deletion and/or *SNF1* overexpression. As expected, the final SAM yield of the mutant Y*REG1*ΔP*SNF1* reached 8.28 g/L in a 10-L fermenter, which was 51.6% higher than the yield of the parent strain *S. cerevisiae* CGMCC 2842.

**Conclusion:**

This study showed that the multiple effects of *REG1* deletion and *SNF1* overexpression improved SAM production in *S. cerevisiae*, providing new insight into the application of the SNF1 complex to abolish glucose repression and redirect carbon flux to nonethanol products in *S. cerevisiae*.

## Background

S-adenosyl-l-methionine (SAM, also named AdoMet), well known as an important bioactive molecule, participates in numerous biological processes, including the regulation of gene expression, metabolism reactions, and signal transduction. Therefore, it has been used in the clinical treatment of liver disorders, depression, osteoarthritis, and Alzheimer’s disease since it was first described by Cantoni in 1952 [1–3]. Further works have been carried out to improve the microbial production of SAM *via* the application of strategies such as strain breeding, fermentation process control and optimization, metabolic engineering, and genome-scale engineering [4, 5].

Currently, various microbes with superior SAM production levels, including *Pichia pastoris* (*P. pastoris*), *Saccharomyces cerevisiae* (*S. cerevisiae*), *Candida utilis*, *Kluyveromyces lactis*, *Escherichia coli* and *Corynebacterium glutamicum*, have been screened for use in the microbial production of SAM [6–9]. Yeast is able to accumulate large amounts of SAM because yeast vacuoles contain large amounts of negatively charged polyphosphate, and SAM has a positive charge [9, 10]. In addition, considering that yeast is generally recognized as a safe microorganism, it is often used as a preferred microbial cell factory for the industrial production of SAM or other target products [5, 10]. Shiozaki et al. reported that a yeast strain *Saccharomyces sake* K26, isolated from more than 300 strains, was able to accumulate 10.8 g/L SAM in a 10-L bioreactor after the optimization of the fermentation medium [11, 12]. Choi et al. overexpressed *SAM2*, which encodes SAM synthase from *S. cerevisiae*, in the leucine auxotroph *Saccharomyces sake* K6-1, leading to increased SAM production levels of 2.8 g/L [13]. Huang et al. reported that the production of 9.64 g/L SAM in fermenter cultivation was achieved by a strain of *S. cerevisiae* through multiple methods, including spaceflight culture, MAT overexpression and optimization cultivation [14]. In our previous studies, a highly SAM-productive yeast strain, named as *S. cerevisiae* CGMCC 2842 (2842), exhibited a high ethionine-resistance and was screened out by mutagenic with ultraviolet irradiation (UV) coupled with ethionine-resistant screening procedure. In the ethionine resistant strain 2842, SAM accumulation occurs by an increase in the methionine resistance [41]. Although the fermentation performance of strain 2842 was degraded after years of passage attributed to defects in genetic stability of ultraviolet mutagenesis breeding, it still had a certain potential for the SAM yield. Chen et al. (2021) reported that the SAM yield from *S. cerevisiae* reached 8.86 g/L in a 10-L bioreactor after deleting *KCS1* (encodes inositol pyrophosphate kinase) and *MLS1* (encodes malate synthase) combined with coexpression of *ACS2* (encodes acetyl-CoA synthase) and *MetK1* (encodes SAM synthase) in the strain 2842 [15].

**Fig. 1:**
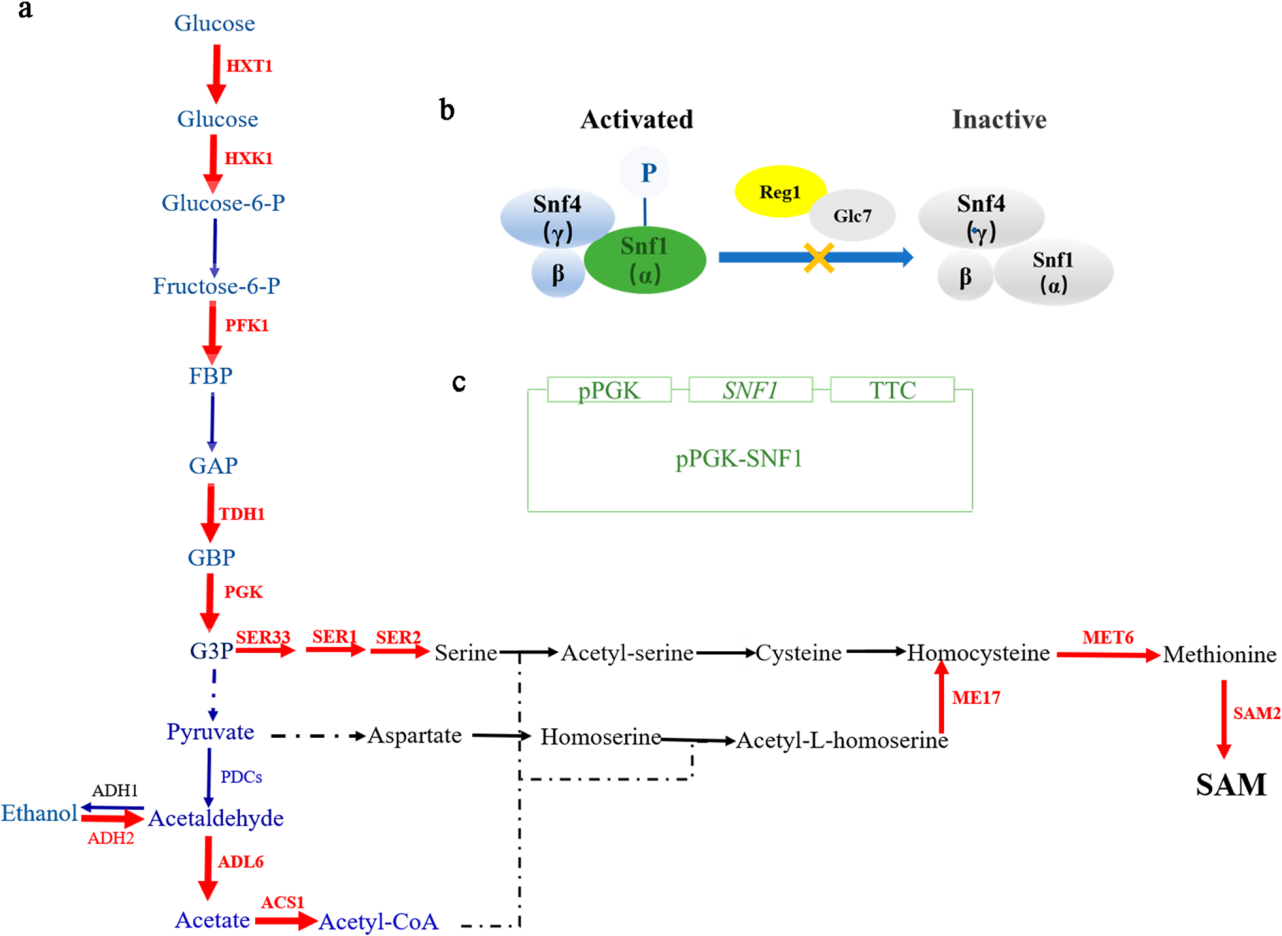
Schematic representation of improving the production of SAM by *REG1* deletion and *SNF1* overexpression in *Saccharomyces cerevisiae*. **a** The glycolytic intermediates are linked to SAM metabolism; **b** The SNF1 complex is inactivated through the dephosphorylation by the type 1 protein phosphatase (PP1) complex Glc7/Reg1. **c** Schematic representation of the recombinant expression vector of *SNF1*. The color of red represents the metabolism enhanced by *REG1* deletion and *SNF1* overexpression; The color of green represents the *SNF1* was overexpressed. The yellow cross represents *REG1* deletion

However, *S. cerevisiae* is a Crabtree-positive yeast, and it undergoes fermentative metabolism when glucose is available in excessive levels, even in the presence of oxygen [16]. Due to oxidative fermentation, *S. cerevisiae* produces a large amount of ethanol in the presence of excessive glucose levels, which leads to the loss of carbon metabolic fluxes, cofactors and energy required for cell growth and SAM production [17, 18]. Continued efforts and different strategies have been undertaken to reduce or eliminate the accumulation of ethanol in the fermentation broth. Lin et al. (2004) reported that the yields of SAM and glutathione from *S. cerevisiae* strain ZJUS1 were increased by 23% and 8.6%, respectively, by reducing the production of ethanol through an appropriate glucose feeding strategy [18]. It is well known that the ethanol synthesis pathway in *S. cerevisiae* mainly involves pyruvate decarboxylases (PDCs, encoded by the *PDC1*, *PDC5* and *PDC6* genes) and ethanol dehydrogenases (ADHs, encoded by the *ADH1*, *ADH2*, *ADH3*, *ADH4*, *ADH5* and *ADH6* genes) (Fig. 1a). Previous studies demonstrated that either deleting ethanol dehydrogenase genes or deleting pyruvate decarboxylase genes completely prevented the production of ethanol but also led to severe physiological defects in yeast [16, 17, 19, 20]. For example, PDC-deficient strains cannot grow with glucose as the sole carbon source, and ADH-deficient strains exhibit weaker growth because of the accumulation of glycerol and acetaldehyde [17, 20, 21]. How to abolish the Crabtree effect in *S. cerevisiae* and redirect carbon flux to nonethanol products is a challenge for the industrial microbial production of SAM and the other target products.

In *S. cerevisiae*, the SNF1 complex, an evolutionarily highly conserved serine/threonine kinase, is composed of a catalytic subunit Snf1 (encoded by the *SNF1* gene), one of the three subunits (Gal83, Sip1 and Sip2) and a regulatory subunit Snf4, and it participates in the global regulation of numerous cellular activities, including glucose repression, stress responses and proliferation [22–24]. The SNF1 complex in yeast is inactivated through the dephosphorylation of the Snf1 subunit by the type 1 protein phosphatase (PP1) complex Glc7/Reg1 (encoded by gens of *GLC7* and *REG1*) when glucose is present at high concentrations [25, 26] (Fig. 1b). When active, the SNF1 complex phosphorylates and deactivates the glucose-induced transcriptional repressor Mig1 and thereby prevents Mig1 from binding to the upstream regulatory element of the genes that encode the transcription activator Sip4 and Cat8, which activate the expression of glucose-repressed genes (Fig. 1b) [23, 27]. It was also reported that the overexpression of the *SNF1* gene enhanced the responses to various environmental stresses, and the overexpression of *SNF4* and the deletion of *GLC7* or *REG1* effectively enhanced maltose metabolism and leavening ability by ameliorating glucose repression in yeast [24, 28, 29].

As reported previously, type 2 alcohol dehydrogenase (ADH2, encoded by the *ADH2* gene), type 6 aldehyde dehydrogenase (ALD6, encoded by the *ALD6* gene) and type 1 acetyl-CoA synthase (ACS1, encoded by the *ACS1* gene) cooperate to catalyze the conversion of ethanol to acetyl-CoA in yeast [27, 30, 31]. The expressions of *ADH2*, *ALD6* and *ACS1* is repressed under conditions of excessive glucose levels in the absence of the active SNF1 complex, and the SNF1 complex is maintained in an inactive state by the active Glc7/Reg1 complex in the presence of glucose [16, 27]. In short, the SNF1 complex, plays a key role for the derepression of structural genes which are repressed in the presence of a high glucose concentration. In this study, it was hypothesized that inducing the expression of *ADH2*,*ALD6* and *ACS1* would reduce the accumulation of ethanol in the fermentation broth, in turn improving the production of SAM. Based on that, a strategy of alleviating glucose repression and reducing the accumulation of ethanol by the *REG1* deletion and *SNF1* overexpression was investigated to improve SAM production in the yeast strain 2842 (Fig. 1b, c). Subsequently, SAM accumulation, growth conditions, glucose consumption, ethanol accumulation, amino acid metabolism, and lifespan, as well as the expression or activities of several enzymes involved in glycolysis and amino acid metabolism, were analyzed in the mutants to reveal that *REG1* deletion combined with *SNF1* overexpression improved the synthesis of SAM.

## Results

### *REG1* deletion and *SNF1* overexpression improved the production of SAM as well as the Dry cell weight (DCW) in *S. cerevisiae*

**Fig. 2:**
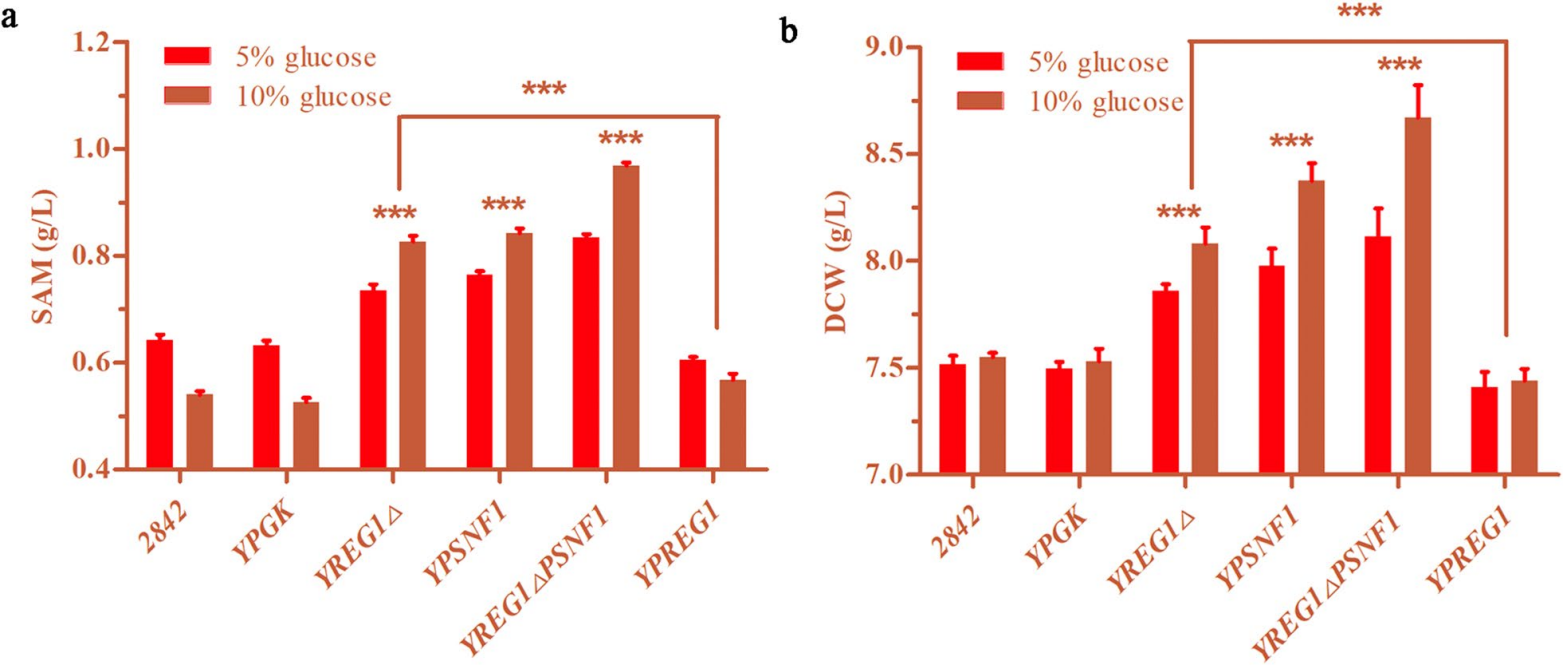
*REG1* deletion and *SNF1* overexpression improved the production of SAM as well as DCW in *S. cerevisiae* in the cultures with 5% and 10% glucose. **a** The SAM production of the mutant strains. **b** The DCW of the mutant strains was significantly improved

**Fig. 3:**
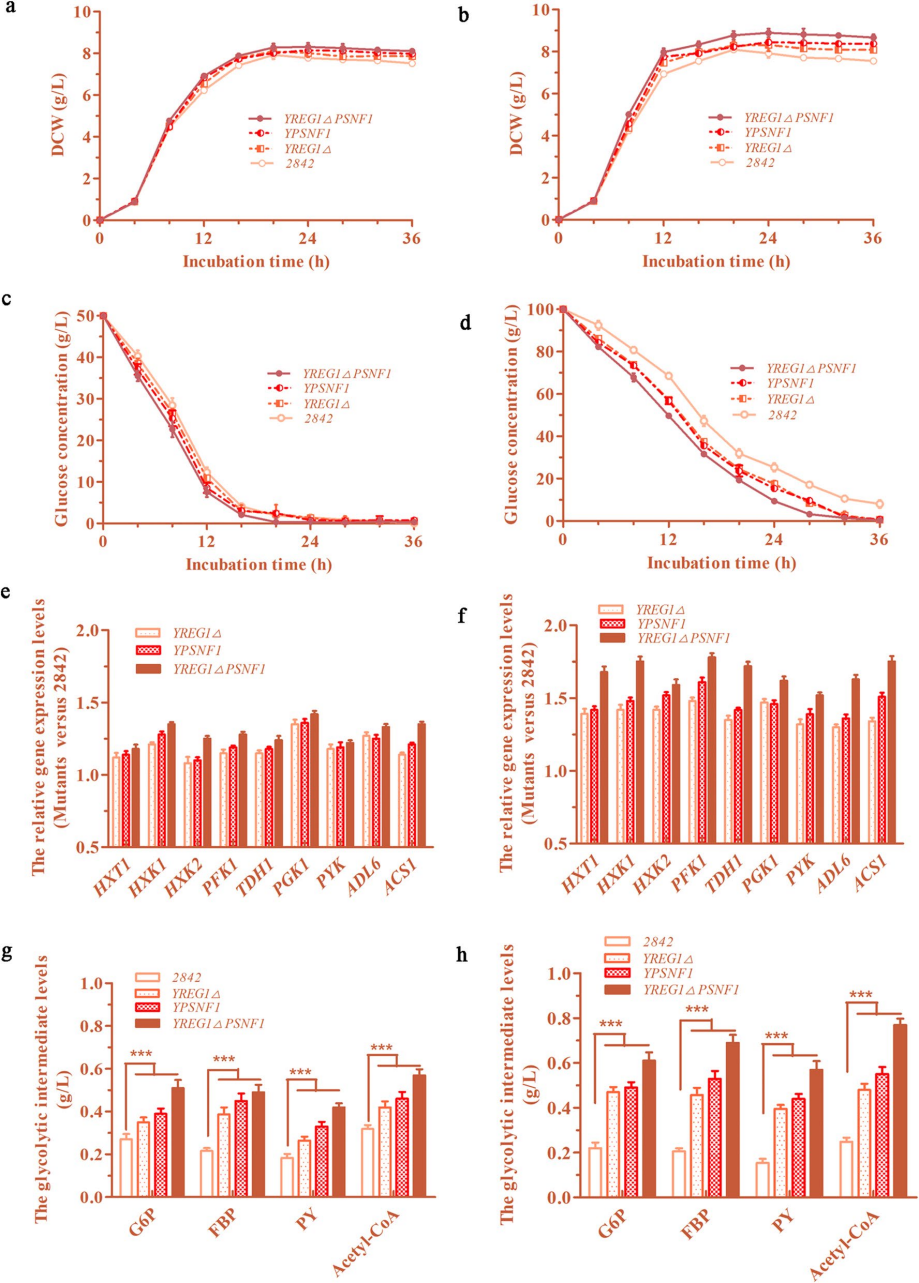
The effects of *REG1* deletion and *SNF1* overexpression to the cell growth, the glucose utilization and the glycolysis in *S. cerevisiae*. **a**, **b** The growth curves of the mutant strains in groups with 5% glucose and 10% glucose, respectively. **c**, **d** The glucose consumptions of the mutant strains in groups with 5% glucose and 10% glucose, respectively. **e**, **f** The relative expression levels of several genes related to glucose transport and glycolytic pathways of the 5% glucose group and the 10% glucose group. **g**, **h** The intracellular levels of several glycolytic intermediates of the groups with 5% glucose and 10% glucose, respectively

To assess the effects of *REG1* and *SNF1* on the production of SAM in yeast, mutants in which *REG1* was deleted and/or *SNF1* was overexpressed were constructed and cultured in medium with different glucose concentrations (5% and 10% glucose). Then, SAM production and DCW in the culture were analyzed. In the group with 5% glucose, the SAM production by the mutant strains Y*REG1*Δ, YP*SNF1* and Y*REG1*ΔP*SNF1* reached 0.73 g/L, 0.76 g/L and 0.83 g/L, which were 14.0%, 18.8% and 29.7% greater than the SAM production by the 2842 strain (produced 0.64 g/L SAM), respectively. In the group with 10% glucose, the SAM production of the Y*REG1*Δ, YP*SNF1* and Y*REG1*ΔP*SNF1* mutant strains reached 0.83 g/L, 0.84 g/L and 0.97 g/L, which were 53.7%, 55.6% and 79.6% greater than the SAM production of the 2842 strain (produced 0.54 g/L SAM), respectively (Fig. 2a). It was demonstrated that the SAM production by the mutant strains was significantly improved compared to that of the wild yeast strain 2842 and the blank control strain YPK, especially in the presence of high concentrations of glucose. Notably, the SAM production of the 2842 strain and the YPK strain under high concentrations of glucose was lower than that at low concentrations of glucose. This means that the parent strain could not adapt to medium with a high level of glucose to produce SAM (Fig. 2a). Similar to the production of SAM, the DCW of the mutant strains was significantly improved compared to that of the wild yeast strain 2842 and the blank control strain YPK (Figs. 2b, 3a and b). To further confirm the impact of *REG1* knockout, *REG1* was reintroduced into the mutant strain Y*REG1*Δ, which led to a decrease in SAM production as well as the DCW (Fig. 2a, b). These data demonstrated that *REG1* deletion and *SNF1* overexpression improved the DCW as well as the production of SAM in *S. cerevisiae*, especially in medium with a high level of glucose.

### *REG1* deletion and *SNF1* overexpression enhanced the glucose utilization due to the enhancement of the glycolysis pathway in *S. cerevisiae*

The glucose utilization and glycolytic responses to *REG1* deletion and *SNF1* overexpression in the strains that produced high levels of SAM were investigated. Compared to the wild-type strain 2842, the mutant strains Y*REG1*Δ, YP*SNF1* and Y*REG1*ΔP*SNF*1 were able to consume glucose in the medium more rapidly, especially when the medium contained high levels of glucose (Fig. 3c, d). To explore the reasons for the increase in glucose consumption, the relative expression levels of several genes related to glucose transport and glycolytic pathways were also analyzed, as shown in Fig. 3e, f. The real-time PCR results showed that the relative expression levels of *HXT1* (encodes the hexose transporter, HXT1), *HXK1* (encodes the hexokinase 1, HXK1), *HXK2* (encodes the hexokinase 2, HXK2), *PFK1* (encodes the 6-phosphofructokinase 1, PFK1), *TDH1* (encodes the glyceraldehyde-3-phosphate dehydrogenase, TDH1), *PGK1* (encodes the 3-phosphoglycerate kinase, PGK1), *PYK* (encodes the pyruvate kinase, PYK), *ALD6* and *ACS1* were significantly upregulated by the *REG1* deletion and the *SNF1* overexpression in the presence of either 5% or 10% glucose (Figs. 1a, 3e and f); the expression levels of these genes in the Y*REG1*ΔP*SNF1* strain were significantly higher than those in the Y*REG1*Δ and YP*SNF1* strains, except for the expression of the *HXT1* and *PYK* genes in the group treated with 5% glucose. This result may have occurred the concentration of glucose decreased at the end of the logarithmic growth phase in the 5% group. It was demonstrated that *REG1* deletion and *SNF1* overexpression effectively enhanced the glycolysis pathway and increased glucose utilization, which were probably beneficial for the production of SAM in yeast.

Quantitative measurements of the intracellular levels of several glycolytic intermediates were further carried out, as shown in Fig. 3g, h. In the 5% glucose group, the intracellular levels of glucose-6-phosphate (G6P) in the Y*REG1*Δ, YP*SNF1* and Y*REG1*ΔP*SNF1* strains reached 0.35 g/L, 0.39 g/L and 0.51 g/L, which were 29.6%, 44.4% and 88.9% higher than that in the 2842 strain, respectively. The levels of fructose-1,6-bisphosphate (FBP) in the Y*REG1*Δ, YP*SNF1* and Y*REG1*ΔP*SNF1* strains reached 0.39 g/L, 0.45 g/L and 0.49 g/L, which were 77.3%, 104.5% and 122.7% higher than that in the 2842 strain (0.22 g/L). Similarly, the levels of pyruvate (PY) in the Y*REG1*Δ, YP*SNF1* and Y*REG1*ΔP*SNF1* strains were increased by 50%, 83.3% and 133.3%, respectively, and the levels of acetyl-CoA in the Y*REG1*Δ, YP*SNF1* and Y*REG1*ΔP*SNF1* strains were increased by 31.3%, 43.8% and 78.1%, respectively (Fig. 3g). The same trend was observed in the 10% glucose group (Fig. 3h). The results showed that the intracellular levels of G6P, FBP, PY and acetyl-CoA were also significantly elevated by *REG1* deletion and *SNF1* overexpression. To further demonstrate the improvement of glucose utilization, the yield of the target product SAM to glucose and the yield of the target product SAM to DCW were calculated as shown in Table 1. It was found that both the yield of the target product SAM to glucose and the yield of the target product SAM to DCW were significantly improved by *REG1* deletion and *SNF1* overexpression (Table 1). In short, *REG1* deletion and *SNF1* overexpression enhanced the glycolysis pathway and improved the glucose utilization, thus increasing the intracellular levels of glycolytic intermediates necessary for yeast cell growth and amino acid biosynthesis.

**Table 1:** The deletion of *REG1* or/and overexpression of *SNF1* increased the glucose utilization and improved the SAM production in yeast in 10% glucose

	2842	YPGK	Y*REG1*△	YP*SNF1*	Y*REG1*△P*SNF1*
SAM (g L^− 1^)	0.54 ± 0.007	0.53 ± 0.008	0.83 ± 0.01***	0.84 ± 0.008***	0.97 ± 0.006***
DCW (g L^− 1^)	7.55 ± 0.02	7.53 ± 0.06	8.08 ± 0.07***	8.38 ± 0.08***	8.67 ± 0.15***
SAM yield to DCW (mg g^− 1^)	71.5	70.4	102.7***	100.2***	111.9***
SAM yield to glucose (mg g^− 1^)	5.4	5.3	8.3***	8.4***	9.7***

### The effects of *REG1* deletion and *SNF1* overexpression on the ethanol oxidation of *S. cerevisiae*

**Fig. 4:**
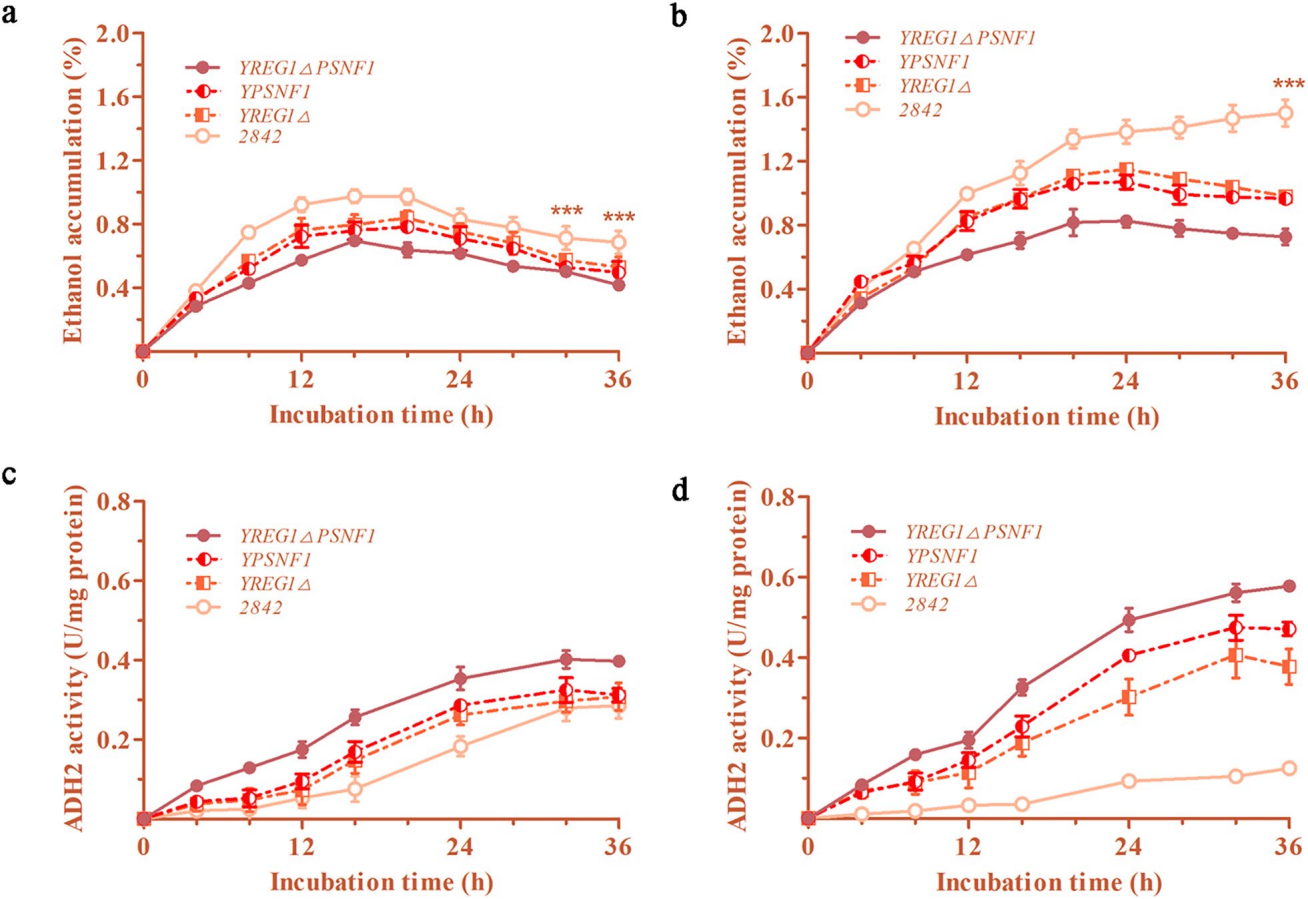
The effects of *REG1* deletion and *SNF1* overexpression on the ethanol oxidation of *S. cerevisiae*. **a**, **b** The ethanol accumulations in the fermentation broth of the mutants in groups with 5% and 10% glucose. **c**, **d** The ADH2 activities of the mutants in groups with 5% and 10% glucose

The accumulation of ethanol and the enzyme activity of ADH2 in fermentation broth were both monitored every 4 h. In the 5% glucose group, the maximum ethanol levels that accumulated in the cultures of the Y*REG1*Δ, YP*SNF1* and Y*REG1*ΔP*SNF1* mutants reached 0.84%, 0.78% and 0.70%, which were 14.2%, 20.4% and 28.6% lower than that in the culture of the 2842 strain, respectively. Thereafter, the ethanol levels in the fermentation broth began to decline slowly, which occurred because the glucose in the fermentation broth was depleted (Fig. 4a). Similarly, in the 10% glucose group, there was a more significant decrease in the ethanol levels in the fermentation broth of the Y*REG1*Δ, YP*SNF1* and Y*REG1*ΔP*SNF1* strains compared with that in the fermentation broth of the 2842 strain. It should be noted that the ethanol accumulation of the wild-type 2842 strain still slowly increased after 36 h of fermentation in 10% glucose, which was due to a large amount of glucose in the culture had not yet been consumed (Figs. 3d, 4b).

Activated ADH2 catalyzes the first step of ethanol oxidation. The ADH2 activity levels are shown in Fig. 4c, d. Overall, the ADH2 activity levels of the Y*REG1*Δ, YP*SNF1*, and especially Y*REG1*ΔP*SNF1* strains, were significantly higher than those of the 2842 strain. However, there were some details in the trends that are worth noting. The ADH2 activity of the 2842 strain was low as long as there was glucose present in the medium, and once the glucose was depleted, the ADH2 activity of the 2842 strain began to increase (Fig. 4c). However, the ADH2 activity levels of the mutants, especially of the Y*REG1*ΔP*SNF1* strain, were significantly higher than that of the 2842 strain regardless of the presence or absence of glucose in the medium. In addition, the relative transcription level of *ALD6* and *ACS1* was also upregulated (Fig. 3e, f), which resulted in a significantly elevated intracellular level of acetyl-CoA (Fig. 3g, h). These data demonstrated that deleting *REG1* and overexpressing *SNF1* did work well to enhance the activity levels of ADH2 and the expression level of *ALD6* and *ACS1*, which are responsible for catalyzing the oxidation reaction of ethanol to acetyl-CoA in *S. cerevisiae*. The decrease in ethanol accumulation means that more carbon sources were probably directed to the synthesis of SAM or other metabolites and to the growth of yeast.

### *REG1* deletion and *SNF1* overexpression enhanced precursor amino acid biosynthesis in *S. cerevisiae*

**Fig. 5:**
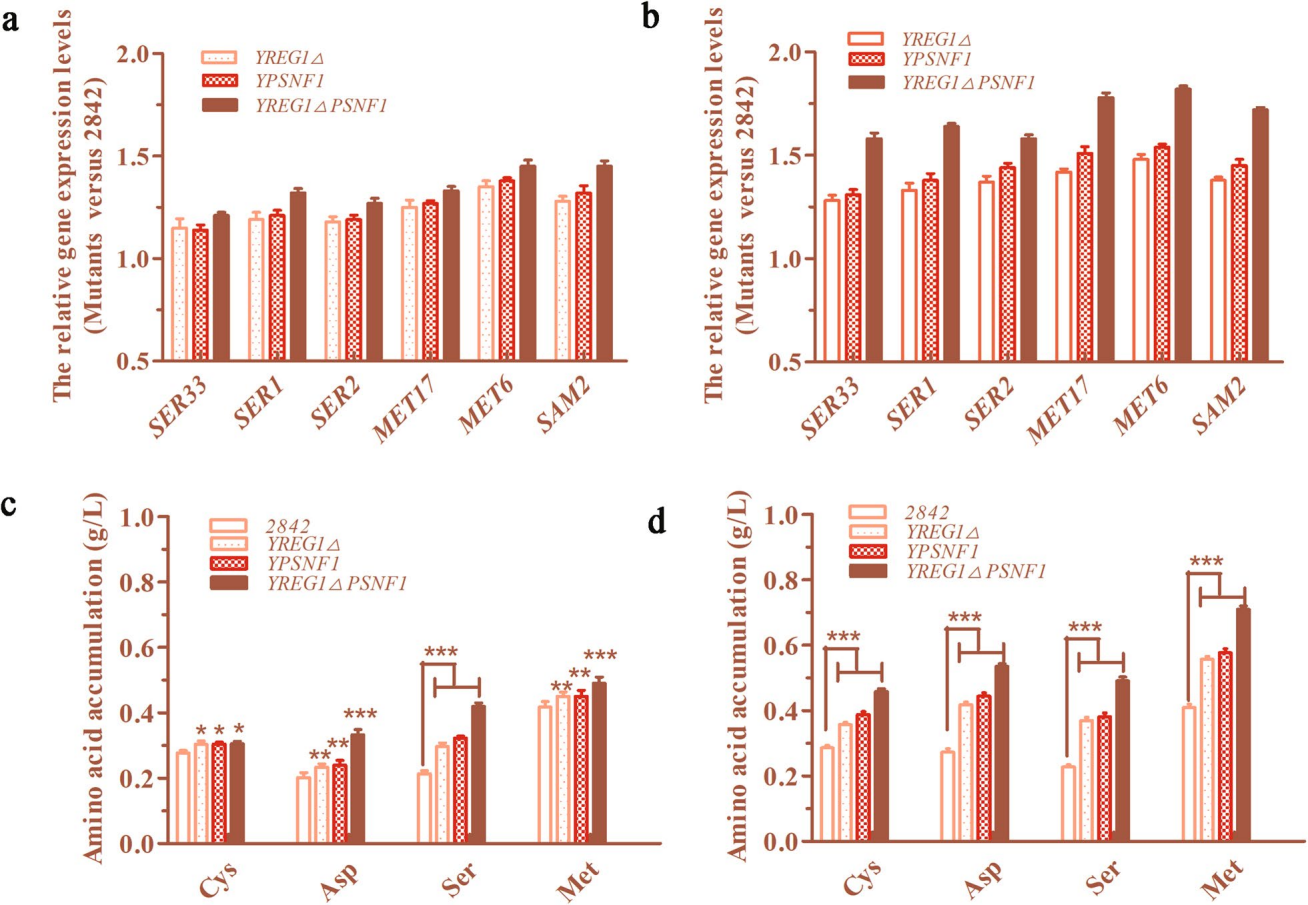
*REG1* deletion and *SNF1* overexpression enhanced precursor amino acid biosynthesis in *S. cerevisiae*. **a**, **b** The relative expression levels of several genes related to amino acid metabolism of the mutants in groups with 5% and 10% glucose. **c**, **d** The precursor amino acids accumulations of the mutants in groups with 5% and 10% glucose

As previously reported, serine, aspartic acid and sulfur-containing amino acids are metabolically related [15, 32]. Therefore, the transcription levels of several genes related to the metabolism of these amino acids were quantitatively analyzed by real-time PCR. It was found that the transcription levels of *SER33*, *SER1*, *SER2*, *MET17*, *MET6* and *SAM2* were obviously upregulated by *REG1* deletion and *SNF1* overexpression. Notably, the transcription levels of *SER33* (encodes phosphoglycerate dehydrogenase, SER33), *SER1* (encodes phosphoserine transaminase, SER1), *SER2* (encodes phosphoserine phosphatase, SER2), MET17 (encodes O-acetyl-homoserine sulfhydrylase, MET17), *MET6* (encodes methionine synthase, MET6), and *SAM2* (encodes SAM synthase 2, SAM2) in the mutant Y*REG1*ΔP*SNF1* strain were 21%, 32%, 27%, 33%, 45% and 45% higher than those in the 2842 strain when cultured in 5% glucose, and these levels were 58%, 64%, 58%, 78%, 82% and 72% higher than those in the 2842 strain in when cultured in 10% glucose (Fig. 5a, b). SER33, SER1 and SER2 together catalyze the conversion of 3-phosphoglycerate (G3P) to serine (Ser), which is a key node in the biosynthesis of sulfur-containing amino acids. As a result of the upregulation of the transcription levels of *SER33*, *SER1*, *SER2*, *MET17*, *MET6* and *SAM2*, the intracellular levels of Ser, l-aspartic acid (Asp), l-cysteine (Cys) and L-methionine (Met), which are precursor amino acids of SAM, were also increased (Fig. 5c, d).

### The lifespan of *S. cerevisiae* was extended by *REG1* deletion and *SNF1* overexpression

**Fig. 6:**
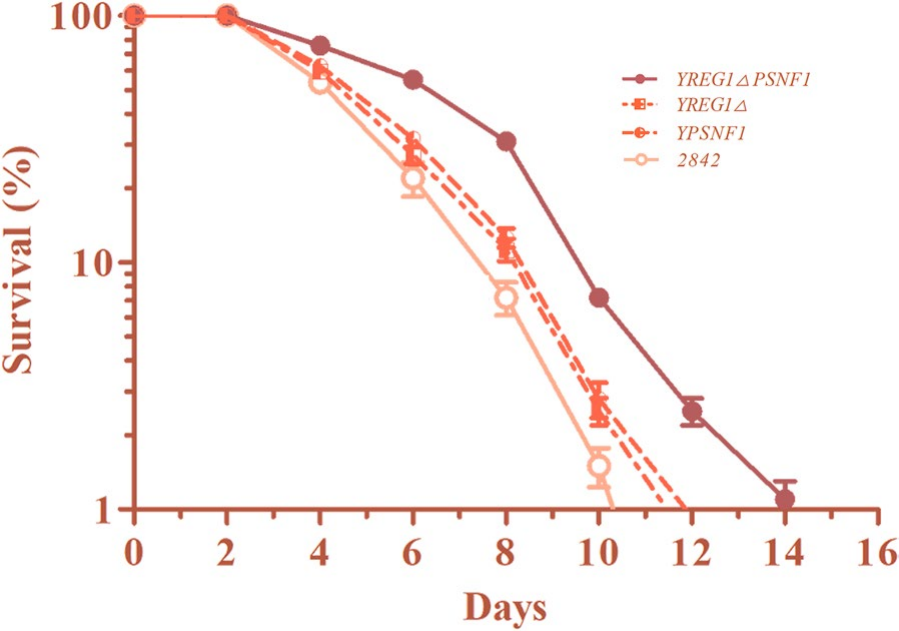
The lifespan of *S. cerevisiae* was extended by *REG1* deletion and *SNF1* overexpression

More cells maintain a longer lifespan in the fermentation broth, which is beneficial for cell factory applications. The activity of SNF1 complex is critical for the extension of the “chronological lifespan” (CLS) of yeast [33]. Therefore, the CLS of *S. cerevisiae* was investigated, and the results are shown in Fig. 6. It was observed that the CLS of Y*REG1*Δ, YP*SNF1* and especially Y*REG1*ΔP*SNF1* was obviously extended compared to that of the wild-type strain 2842. In addition, the strain in which *REG1* was deleted and *SNF1* was overexpressed had a longer lifespan extension than that the Y*REG1*Δ or YP*SNF1* strains. The results of our research are consistent with the findings of Ogawa et al., who reported that stimulating SAM synthesis extends the yeast lifespan *via* the activation of the universal energy-sensing regulator Snf1 [34].

### The SAM accumulation capacity of the mutant strain Y *RGE1* ΔP *SNF1* by fed-batch fermentation

**Fig. 7:**
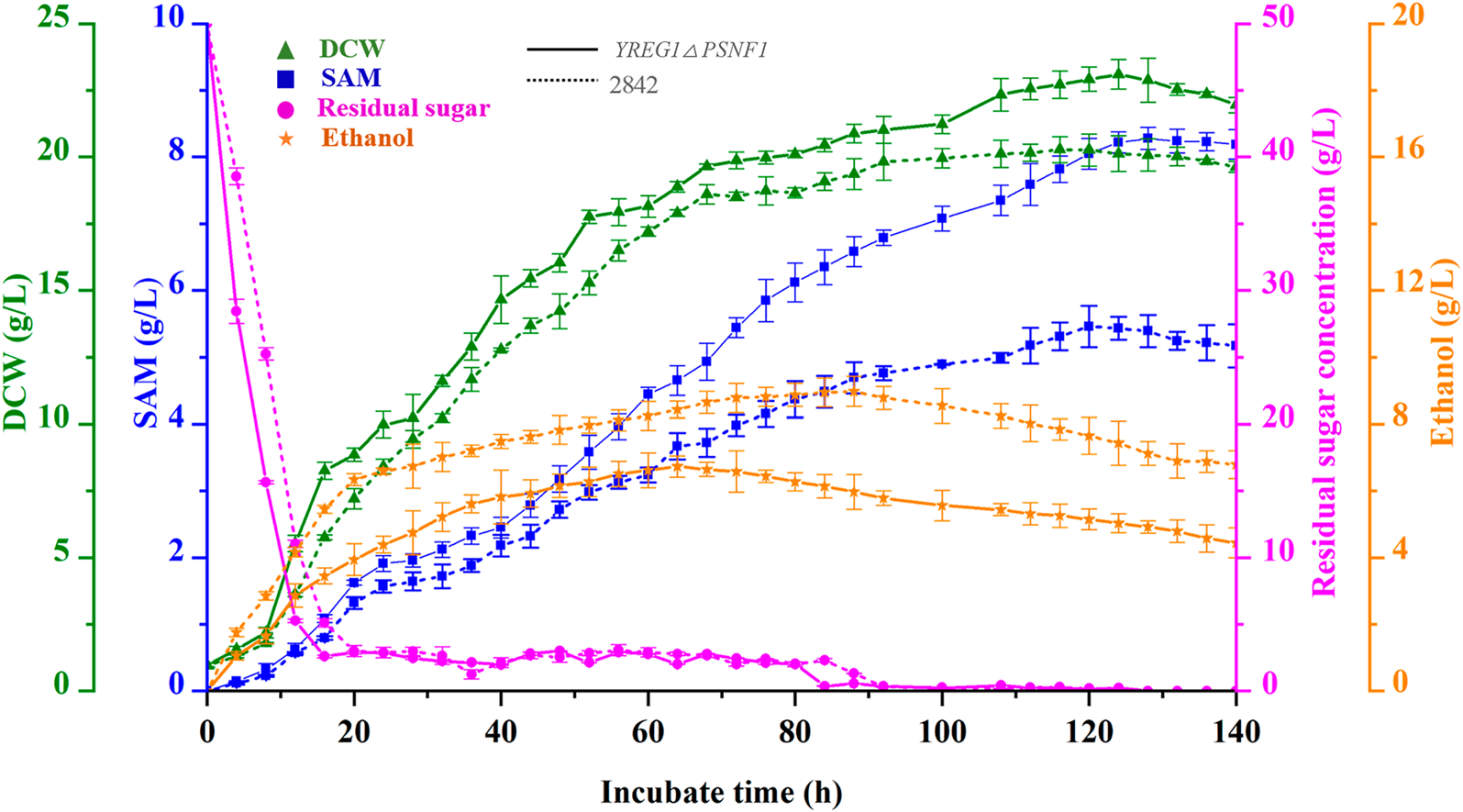
The SAM accumulation capacity of the mutant strain Y*RGE1*ΔP*SNF1* by fed-batch fermentation

To assess the SAM production capacity of the mutant strain Y*REG1*ΔP*SNF1*, a preliminarily scaled-up fed-batch fermentation experiment was carried out in a 10-L fermentation tank, and SAM production compared with that of the SAM-producing *S. cerevisiae* 2842 strain, according to our previous study [15, 35]. SAM production, ethanol accumulation, glucose consumption and DCW were analyzed during the whole fed-batch fermentation process (Fig. 7).

It was found that the mutant strain Y*REG1*ΔP*SNF1* was able to consume glucose slightly more rapidly than the 2842 strain. When the concentration of residual sugar was lower than 5 g/L, to enhance the biomass and maximize SAM production, molasses was fed into the fermentation broth at a rate of 1.2 g/L/h for 72 h. As we expected, the ethanol levels in the fermentation broth of the mutant strain Y*REG1*ΔP*SNF1* were obviously decreased, and the maximum ethanol level in the fermentation broth of the mutant strain Y*REG1*ΔP*SNF1* was 6.73 g/L at 64 h, which was 25% lower than that in the fermentation broth of the wild-type 2842 strain (8.98 g/L). As a result, the growth vitality was maintained, and there was a slight increase in the biomass, with the maximum DCW of 23.1 g/L at 124 h. The SAM production of the mutant strain Y*REG1*ΔP*SNF1* was ultimately enhanced, with a maximum SAM production of 8.28 g/L at 128 h, which represented an increase of 51.6% compared with that of the parent strain 2842 (Fig. 7). The above data showed that the strategy of *REG1* deletion combined with *SNF1* overexpression exhibited great potential for improving the production of SAM in yeast.

## Discussion

The SNF1 complex, a well-known global regulatory factor, plays key roles in the regulation of gene transcription, metabolism, stress response, cell aging, and so on. Although its structure is well known, research on its various cellular regulatory mechanisms and their potential applications is ongoing. For example, the SNF1 complex has been applied to improve yeast cell resistance to various environmental stresses, improve lipid accumulation, improve leavening abilities, and so on [24, 26, 29]. In this study, the multiple effects of *REG1* deletion combined with *SNF1* overexpression were used to improve SAM production in *S. cerevisiae*, which provided a reference for the application of the SNF1 complex to abolish glucose repression and redirect carbon flux to nonethanol products in *S. cerevisiae*.

The responses of glucose utilization to *REG1* deletion combined with *SNF1* overexpression were investigated. It was shown that the *REG1* deletion combined with the *SNF1* overexpression enhanced the glucose utilization of the *S. cerevisiae*, which occurred to several following reasons. On the one hand, the transcription levels of genes involved in the processes of the glucose transport (*HXT1*) and glycolysis (for example, the *HXK1*, *HXK2*, *PFK1*, *TDH1*, *PGK1*, and *PYK* genes), were elevated by *REG1* deletion combined with *SNF1* overexpression, especially when grown in a higher concentration of glucose. On the other hand, *SNF1* overexpression improved cell tolerance, such as tolerance to heat, high glucose or ethanol, and it could also improve the utilization of glucose, which was reported by Meng et al. [36]. It was interesting to note that our results do not seem to be consistent with the description by Meng et al. regarding the effect of *SNF1* overexpression on the growth of yeast at different glucose concentrations. Meng et al. reported that *SNF1* overexpression could exert negative effect on the growth of yeast in the presence of 7% glucose, in contrast, this genetic manipulation significantly improved the cellular growth of the recombinant yeast (Fig. 2b) under comparable glucose conditions (5% and 10%) in this study. Several reasons, for example, differences in genetic background and the culture conditions, probably account for this discrepancy, and it is likely to be a new subject that deserves further attention.

The third reason for the enhancement of carbon resource utilization should be the reduction of ethanol accumulation in the fermentation broth. In *S. cerevisiae*, there are 5 types of alcohol dehydrogenases (ADHs). The ADH1, ADH3, ADH4 and ADH5 proteins are responsible for reducing acetaldehyde into ethanol during glucose fermentation. In the presence of glucose, the expression of *ADH2* is repressed by the absence of active SNF1, which is deactivated by the Glc7/Reg1 complex [27, 37]. When glucose is depleted from the medium, the expression of the *ADH2* gene is increased to produce ADH2 for ethanol oxidation. The derepression of the *ADH2* gene requires the synergistic activators Adr1 and Cat8 [38]. The SNF1 complex regulates both the expression and the activity of Cat8 [31, 39]. In this study, it was found that *REG1* deletion combined with *SNF1* overexpression decreased ethanol accumulation in the fermentation broth. Another piece of evidence of ethanol oxidation in the fermentation broth is the increased expressions of the *ALD6* gene the *ACS1* gene. The reduction of ethanol accumulation in the fermentation broth is beneficial both for cell growth and for the accumulation of metabolites such as SAM.

These data demonstrated that *REG1* deletion combined with *SNF1* overexpression enhanced glucose transport and the glycolytic pathway. An enhancement in the glycolytic pathway will most likely affect the intracellular levels of glycolytic intermediates. The levels of several glycolytic intermediates were indeed significantly enhanced, as shown in Fig. 3g, h. It is well known that the glycolytic pathway provides precursors for the synthesis of several amino acids [15, 40]. Therefore, the relative transcription levels of *SER33*, *SER1*, *SER2*, *MET17*, *MET6* and *SAM2* were quantitatively analyzed and were significantly upregulated by *REG1* deletion combined with *SNF1* overexpression. SER33, SER1 and SER2 together catalyze the conversion of G3P to Ser, which is a key node in the biosynthesis of sulfur-containing amino acids. MET17, MET6 and SAM2 are responsible for the synthesis of homocysteine, Met and SAM (Fig. 1a) [32]. As expected, the intracellular levels of Ser, Asp, Cys and Met, which are precursor amino acids for the biosynthesis of SAM, were ultimately increased. The response of amino acid biosynthesis to *SNF1* overexpression was consistent with a previous report [36].

SAM has received much attention as a clinical therapy for many diseases, such as liver disorders, depression, osteoarthritis, and Alzheimer’s disease. In addition to its role in the regulation of numerous biological processes, including metabolism, signal transduction and gene expression, it was also reported that stimulating SAM synthesis led to enhanced stress resistance and extended the yeast lifespan via the activation of the SNF1 complex. Here, the CLSs of the mutants Y*REG1*Δ, YP*SNF1* and especially Y*REG1*ΔP*SNF1* were obviously extended compared to that of the parent strain 2842; that is, *REG1* deletion combined with *SNF1* overexpression extended the yeast lifespan. The reason why *REG1* deletion and *SNF1* overexpression extended the lifespan is probably related to the increased resistance of the yeast to environmental stress and the increased carbon source utilization; however, more studies are needed on this topic [33, 34, 36]. This study presented evidence that the energy-sensing regulator SNF1 complex together with its up- and downstream regulators may be targets for improving SAM biosynthesis and delaying age-related disorders; this study probably provided new insight into the role of SAM in our health.

## Conclusion

The multiple effects of *REG1* deletion and/or *SNF1* overexpression exhibited a great potential for improving the SAM production in yeast. Enhanced the expression levels of genes involved in glucose transport and glycolysis, which improved the glucose utilization and then elevated the levels of glycolytic intermediates. The expression levels of *ACS1* (encoding acetyl-CoA synthase I) and *ALD6* (encoding aldehyde dehydrogenase), and the activity of alcohol dehydrogenase II (ADH2) were enhanced especially in the presence of excessive glucose levels, which probably promoted the conversion of ethanol in fermentation broth into acetyl-CoA. The gene expressions involved in sulfur-containing amino acids were also enhanced for the precursor amino acid biosynthesis. As expected, the enhanced glucose utilization and ethanol oxidation allowed more carbon flux to be redirected to precursor amino acid biosynthesis and extension of yeast lifespan. This study provides new insight into the application of the SNF1 complex to abolish glucose repression and redirect carbon flux to the production of SAM and other nonethanol products in *S. cerevisiae*.

## Methods

### Strains and plasmids

All the strains, plasmids and primers involved in this study are shown in Table 2. The parent strain of *Saccharomyces cerevisiae* CGMCC 2842 (2842) is a strain that produces high levels of SAM and was obtained from the China General Microbiology Culture Collection Center (Beijing, China) [41]. For the construction of the *REG1* knockout strain, the short flanking homology (SFH) region replacement method was used, as described in previous studies [29, 42]. The gene knockout cassette of loxP-kanMx-loxP was obtained by PCR amplification using pUG6 as the template, and the G418 resistance gene marker was rescued by transforming the plasmid pSH65 with bleomycin resistance into positive transformants and inducing Cre recombinase expression with D-galactose. The primer pairs A & B and C & D were used for the deletion of *REG1*.

**Table 2:** Strains, plasmids and primers

Strains, Plasmids and Primers	Relavant characteristics	Source
*Strains*		
2842	*S. cerevisiae* CGMCC 2842, wild type strain	Cao et al. 2012
Y*REG1*△	*S. cerevisiae* CGMCC 2842 derivative, *REG1*△	This study
YPGK	*S. cerevisiae* CGMCC 2842 derivative, containing pPGK-KanMX	This study
YP*REG1*	*S. cerevisiae* CGMCC 2842 derivative, containing pPGK-*REG1*	This study
YP*SNF1*	*S. cerevisiae* CGMCC 2842 derivative, containing pPGK-*SNF1*	This study
Y*REG1*△P*SNF1*	YREG1△derivative, *REG1*△, containing pPGK-*SNF1*	This study
*Plasmids*		
pYES 2.0	2µ, URA3	Invitrogen
pYES-KanMX	pYES 2.0 derivative, pGAL, 2µ, G418 resisitance gene	Cao et al. 2012
pPGK-KanMX	pYES-KanMX derivative, pPGK, 2µ, G418 resisitance gene	This study
pPGK-*REG1*	pPGK-KanMX derivative, pPGK, 2µ, G418 resisitance, expression of *REG1* of *Saccharomyces cerevisiae*	This study
pPGK-*SNF1*	pPGK-KanMX derivative, 2µ, G418 resisitance, expression of *SNF1* of *Saccharomyces cerevisiae*	This study
pUG6	Template plasmid containing loxP-KanMX-loxP elements	Euroscarf
pSH65	Cre containing plasmid for loxP-KanMX-loxP cassette recycle	Euroscarf
*Primers*		
*pPGK*-F	CCGCTCGAGTATTTTAGATTCCTGACTTC	This study
*pPGK*-R	CGCGGATCCTGTTTTTATATTTGTTGTA	This study
*SNF1*-F	CGC GGATCC ATGAGCAGTAACAACAACAC	This study
*SNF1*-R	CG GAATTC TCAATTGCTTTGACTGTTAAC	This study
*REG1*-F	GGGGTACC ATGTCAACAAATCTAGCAAATTAC	This study
*REG1*-R	CGAGCTC CTAACTGCTGTCATTTCCATTTTC	This study
A	ATGTCAACAAATCTAGCAAATTACTTCGCCGGTAAGAAAG **TACGCTGCAGGTCGACAAC**	This study
B	CTAACTGCTGTCATTTCCCTTTTCTTGTGGCTTGACGTCA **TAGGCCACTAGTGGATCTG**	This study
C	GGCCTTCAGTATCGATGGCTGTTCAAGCGAAAAATGATG **TACGCTGCAGGTCGACAAC**	This study
D	TCTAGTGGTTTGCACGCTATCAACTTTCTTGTCCTTTAGC **TAGGCCACTAGTGGATCTG**	This study

A promoter of *PGK1* (*pPGK*) with *Eco* RI/Not I sequences was amplified by PCR from the genomic DNA of the yeast strain 2842 and was inserted into the corresponding site of pYES-KanMx to obtain pPGK-KanMx. Then, a 3 kb nucleotide fragment including the *REG1* gene with *Not* I/*Sph* I sequences and a 1.9 kb nucleotide fragment including the *SNF1* gene with *Not* I/*Sph* I sequences were amplified by PCR from the genomic DNA of the yeast strain 2842 and then inserted into the corresponding site of pPGK-KanMx to obtain pGAL1-*REG1* and pPGK-*SNF1*, respectively.

The plasmids pPGK-KanMx, pPGK-*REG1*, pPGK-*SNF1* and the gene knockout cassettes were transformed into the parent strain 2842 using the lithium acetate method with G418 resistance selection, as described in previous studies [43]. All of the recombinant mutants were verified by DNA sequencing.

### Media and culture conditions

The media and the culture conditions were previously described [15, 41]. Briefly, the yeasts, preserved in glycerin tubes, were smeared onto fresh YPD plates and incubated at 30 °C for 20 h. The colonies were picked and transferred into 50 mL of YPD medium and incubated for 20 h at 30 °C with shaking at 200 rpm. To maintain the genetic stability of the recombinant plasmids, 250 µg/mL G418 was added to the medium to maintain selection pressure. 5% inoculations were transferred to 50 mL of O-medium and fermented for 48 h at 30 °C with shaking at 200 rpm. The SAM production capacity of the recombinant mutant Y*REG1*ΔP*SNF1* was assessed in a 10-L fermenter containing 7 L of O-medium (B. Braun Biotech, Melsungen, Germany), as previously described [15]. The YPD medium was composed of 20 g/L glucose, 20 g/L yeast extract and 10 g/L peptone. The YPD solid medium contained YPD medium plus 20 g/L agar powder. The O-medium contained 50 g/L glucose or 100 g/L glucose, 5 g/L yeast extract, 10 g/L peptone, 2 g/L K_2_HPO_4_, 4 g/L KH_2_PO_4_, 0.5 g/L MgSO_4_·7H_2_O, and 1.5 g/L L-methionine, pH 6.0 [41].

### DCW and glucose consumption assays

The DCW and glucose consumption were monitored every 4 h throughout the fermentation process. The DCW was measured following the methods reported by Chen et al. [35]. The glucose concentration in the fermentation broth was measured by the 3,5-dinitrosalicylic acid method [44].

### Amino acid analyses

The cells were harvested at 36 h of the fermentation and prepared according to the methods of previous reports for amino acid analyses [15, 41]. The levels of SAM, Met, Ser and Asp were measured by a Shimadzu LC10A HPLC system (Shimadzu, Kyoto, Japan) equipped with a Megres C18 column (5 μm, 4.6 mm × 250 mm) (Hanbon Sci. & Tech., China). Peak area analysis was performed based on the standard calibration curves of SAM, Met, Ser and Asp (Sangon, Shanghai, China) according to the methods of previous reports [15, 44, 45]. For the measurement of Cys, the precolumn derivatization high-performance liquid chromatographic method was performed according to previous reports [46].

### Intracellular glycolytic intermediate measurements

The cells were harvested at 36 h of the fermentation and prepared according to the methods of previous reports for the measurements of G6P, FBP, and PY: 2.0 mL of fermentation broth was centrifuged at 8000 rpm for 10 min, mixed with 2.0 mL of 1.5 mol/L perchloric acid and stored at 40 °C for 30 min. Then, the mixture was centrifuged at 8000 rpm for 5 min to remove proteins, and then, the supernatant was collected. Next, the supernatant was neutralized with 2.5 mol/L K_2_CO_3_ at 40 °C [15, 47]. The intracellular level of G6P was assayed by the procedure reported by Zhu et al. [47]. The FBP assay was performed according to the procedure reported by Du et al. [48]. The intracellular level of PY was assayed enzymatically with lactic acid dehydrogenase according to the method reported by Saavedra et al. [49]. The procedures used to extract acetyl-CoA and measure its levels were performed according to previous reports [35].

### RNA extraction, reverse transcription and real-time qPCR

To determine the expression levels of several glycolytic genes, total RNA was isolated from yeast cells using the Total RNA Isolation Kit (Sangon, Shanghai, China). The RNA quality was verified on a 1% agarose gel, and the concentration was measured with an Eppendorf BioPhotometer Plus (Hamburg, Germany). The same concentration of total RNA (1 µg) from each sample was reverse transcribed using the PrimeScript RT Reagent Kit with gDNA Eraser (Perfect Real Time, Takara) following the manufacturer’s instructions. The primers used to quantitatively analyze the expression of the target genes *HXT1*, *HXK1*, *HXK2*, *PFK1*, *TDH1*, *PGK1*, *PYK*, *ALD6*, *ACS1*, *SER33*, *SER1*, *SER2*, *MET17*, *MET6*, and *SAM2* and the reference gene *ACT1* are listed in Table 3. Gene transcription was quantified by real-time qPCR on a StepOnePlus instrument (ABI, USA) by using the 2^−ΔΔCT^ algorithm [15, 36, 50].

**Table 3:** The primers used in Q-PCR analyses of the gene expression in this study

Gene	Sense primer	Antisense primer
*HXT1*	tgtgccattggtggtatcgt	accaccgacacctaaaccag
*HXK1*	tactggtgtcaacggtgctt	gttcgtcgacagcaacatcg
*HXK2*	ctgctccaatggccatcaac	aaggtttgttggcctggtct
*PFK1*	tggtcttgtcggttccatcg	aaggtttgttggcctggtct
*TDH1*	ttgaggttgttgctgtcaacg	gcttgtcgtcatgggaaacag
*PGK1*	aggcttctgccccaggttc	cagcacgttgtggcaagtc
*PYK*	cgactcagatgctggattca	ccgtttctccagaaagcataa
*ALD6*	gaacttcaccaccttagagcca	gcagcgggtttcaagataca
*ACS1*	gctgcaaaggataaggatgg	gcctcaatttcagcggtaga
*SER33*	ctcgtcgtgtaagcatt	acttgtggaactctactt
*SER1*	acaactcagcctataatacaa	ccaatgcctcatataatatctt
*SER2*	cctatcgtagacggacag	caccatacaacttgcttca
*MET17*	aaatggattggtggtcatgg	gaaagaggcaaatgggttca
*MET6*	tggaagctgccggtatcaag	gcaactctgaaagcttcggc
*SAM2*	tgaatccgtcggtgaaggtc	agctgtttcacaggcaacct
*ACT1*	acgctcctcgtgctgtcttc	gttcttctggggcaactctca

#### Enzymatic assays

The yeast cells were sampled every 4 h and centrifuged at 8000 rpm for 5 min and washed twice with a phosphate-buffered (0.1 mol/L, pH 7.0) solution. Cell-free extracts were prepared by ultrasonication. The ultrasonic conditions were as followed: the total time was 10 min (on-time 10 s and offtime 10 s), the ultrasound power was 300 W at 22 kHz frequency [15]. The determination of ADH2 activity was performed according to the protocols reported by Mauricio et al. and Johansson and Sjöström [51, 52]. The activity ADH2 was measured in cuvettes with 20 µL of crude extract (after incubation at 60 °C for 15 min, for the thermal denaturation of ADH1) and 2.98 mL of reaction mixture, which contained 1 mmol/L NAD^+^, 2 mmol/L β-mercaptoethanol, 70 mmol/L semicarbazide, and 100 mmol/L pyrophosphate buffer (pH 8.5). The reaction was started by the addition of 100 mmol/L ethanol. The changes in absorbance at 340 nm were monitored. One unit of activity was defined as the amount of enzyme that formed 1 µmol NADH per min at 25 °C.

#### Chronological life span assays

The CLS analysis was performed in liquid SDC media (defined below), according to the previously described [33, 34, 53]. The cultures grown overnight were diluted (2 × 10^6^ cells/mL) in SDC media and cultured at 30 °C, 200 rpm. The viability was measured by plating the yeast cells onto the YPD plates and monitoring CFUs starting from day 2, which was considered to be the initial survival (100%). SDC medium contained 2% glucose, 0.5% ammonium sulfate, 0.18% yeast nitrogen base without amino acids and ammonium sulfate, amino acids to a final concentration of 20 mg/L (adenine, arginine, histidine, methionine, tryptophan, and uracil), 30 mg/L (isoleucine, leucine, lysine, and tyrosine), 60 mg/L (phenylalanine), and 150 mg/L (valine).

### Statistical analysis

All the experiments in this study were repeated three times, each time in triplicate. The data represent biological replicates. The statistical analyses were performed with GraphPad Prism software, San Diego California, USA. The error bars correspond to the standard error of the mean of biological replicates. The number of asterisks denotes significant differences between the recombinant strain and the wild-type strain, with p < 0.001 (***), p < 0.01 (**) and p < 0.05 (*).

## Data Availability

All data generated or analyzed during this study are included in this published article.
